# Predicting human splicing branchpoints by combining sequence-derived features and multi-label learning methods

**DOI:** 10.1186/s12859-017-1875-6

**Published:** 2017-12-01

**Authors:** Wen Zhang, Xiaopeng Zhu, Yu Fu, Junko Tsuji, Zhiping Weng

**Affiliations:** 1 School of Computer, Wuhan University, Wuhan, 430072 China; 2 School of Computer Science, Carnegie Mellon University, 5000 Forbes Avenue, Pittsburgh, PA 15213 USA; 3 Program in Bioinformatics and Integrative Biology, University of Massachusetts Medical School, 368 Plantation Street, Worcester, MA 01605 USA

**Keywords:** Genetic algorithm, Multi-label learning, Human splicing branchpoint, Logistic regression

## Abstract

**Background:**

Alternative splicing is the critical process in a single gene coding, which removes introns and joins exons, and splicing branchpoints are indicators for the alternative splicing. Wet experiments have identified a great number of human splicing branchpoints, but many branchpoints are still unknown. In order to guide wet experiments, we develop computational methods to predict human splicing branchpoints.

**Results:**

Considering the fact that an intron may have multiple branchpoints, we transform the branchpoint prediction as the multi-label learning problem, and attempt to predict branchpoint sites from intron sequences. First, we investigate a variety of intron sequence-derived features, such as sparse profile, dinucleotide profile, position weight matrix profile, Markov motif profile and polypyrimidine tract profile. Second, we consider several multi-label learning methods: partial least squares regression, canonical correlation analysis and regularized canonical correlation analysis, and use them as the basic classification engines. Third, we propose two ensemble learning schemes which integrate different features and different classifiers to build ensemble learning systems for the branchpoint prediction. One is the genetic algorithm-based weighted average ensemble method; the other is the logistic regression-based ensemble method.

**Conclusions:**

In the computational experiments, two ensemble learning methods outperform benchmark branchpoint prediction methods, and can produce high-accuracy results on the benchmark dataset.

## Background

Alternative splicing is a regulated event in a single gene coding for proteins. Alternative splicing processes pre-messenger RNAs by removing introns and joining exons [1–3]. Consequently, the alternatively spliced mRNAs are translated as multiple proteins, and exert different functions. The studies show that the alternative splicing may be associated with genetic diseases [4, 5].

For an intron, the alternative splicing is activated by signals from 3′ end of the intron (3SS), 5′ end of an intron (5SS) and branchpoints (BPs). BP site selection is the primary step of the alternative splicing, and causes inclusion of the downstream exon in the mRNA. Branchpoints provide critical information for alternative splicing, and the investigation of branchpoints can help to understand the mechanism of the pre-messenger RNA transcript and the consequent biological events. Researchers discovered branchpoints by wet experiments, but many branchpoints are still unknown and need to be identified. Wet experiments are usually time-consuming, and researchers developed computational methods to guide wet experiments.

In recent years, researchers studied splicing branchpoints and analyzed their characteristics [6, 7]. First, the locations of most BPs are close to 3SS of introns; second, most BPs are adenines; third, dinucleotide “AG” is likely to be depleted between BPs and 3SS. Because researchers have knowledge about branchpoints, the development of computational methods becomes possible. Gooding et al. [8] trained the position weighted matrices by using human branchpoints, and then utilized the matrix to predict putative BPs. Schwartz et al. [9] defined patterns: NNYTRAY, NNCTYAC, NNRTAAC and NNCTAAA, and then scanned 200 nt upstream of 3SS to obtain heptamers which conform to any of these patterns. Heptamers were scored by using the hamming distance to the pattern TACTAAC. Plass et al. [6] obtained nonamers by scanning 100 nt upstream of the 3SS, and then scored nonamers by using entropy between nonamers and the motif “TACTAACAC”. Corvelo et al. [10] compiled positive instances and negative instances by scanning 500 nt upstream of 3SS, and then built BP predictors by using support vector machine.

Although several computational methods have been proposed for the branchpoint prediction, there is still room to improve the prediction performances. One point is that an intron may have more than one branchpoints, and the prediction models should take multiple branchpoint into account. The other point is how to make use of characteristics of introns. First of all, we formulate the original problem as a multi-label learning task, which can deal with multiple BPs in introns. Second, we investigate a variety of intron sequence-based features, including sparse profile, dinucleotide profile, position weight matrix profile, Markov motif profile, and polypyrimidine tract profile. Third, we consider several multi-label learning methods: partial least squares regression [11], canonical correlation analysis [12] and regularized canonical correlation analysis [13] for modelling. Fourth, we design ensemble learning schemes which integrate different features and different classifiers to build BP prediction models. Base predictors and ensemble rules are critical components in the design of ensemble systems. In our previous work [14], we determined a feature subset, and built individual feature-based models by using the feature subset and three multi-label learning methods; the average scores from different models were adopted for predictions. However, diversity of base predictors is limited, and the average scoring strategy is arbitrary. Therefore, we redesign the strategies to build prediction models by combining diverse features and different multi-label learning methods. Here, we generate different feature subsets and combine different multi-label learning methods to build diverse base predictors; we consider two ensemble rules: the weighted average rule based on genetic algorithm optimization and the nonlinear rule based on the logistic regression. Finally, we develop two ensemble models for the branchpoint prediction. One is the genetic algorithm-based weighted average ensemble method; the other is the logistic regression-based ensemble method. In the computational experiments, two ensemble learning methods have high-accuracy performances on the benchmark dataset, and produce better results than other state-of-the-art BP prediction methods. Moreover, our studies can reveal the importance of features in the BP prediction, and provide the guide to the wet experiments.

## Methods

### Dataset

In recent years, Mercer et al. [15] used the technique that combine exoribonuclease digestion and targeted RNA-sequencing [16] to enrich for sequences that traverse the lariat junction, and then identified human branchpoints efficiently. Therefore, they obtained 59,359 high-confidence human branchpoints in more than 10,000 genes, and compiled a detailed map of splicing branchpoints in the human genome. The data facilitate the development of human branchpoint prediction models.

Here, we process Mercer’s data [15]. Specifically, we remove redundant records in which same introns are originally from different genes, and obtain 64,155 unique intron-branchpoint records. In the records, a branchpoint may be responsible for several introns, and an intron may have multiple BPs.

**Fig. 1:**
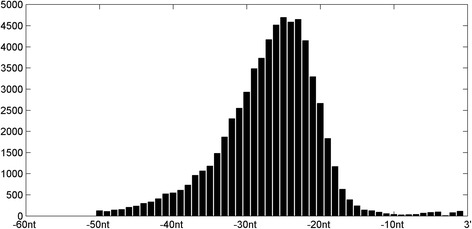
Distribution of branchpoints near to 3′ of introns

Despite introns have long lengths, studies [15] revealed that branchpoints are close to 3SS of introns. The distribution of BP sites in Mercer’s dataset is demonstrated in Fig. 1. According to the distribution, most BPs are located between 50 and 11 nt upstream of 3SS, and 99% intron-branchpoint records (63,371/64,155) fall in this region. Therefore, we pay attention to the branchpoints between 50 and 11 nt upstream of 3SS of introns, and build models to predict BPs located in the region. For this reason, we only use intron sequences and their BPs in specified regions, and compile a benchmark dataset which has 63,371 intron-branchpoint records. The benchmark dataset covers 42,374 introns and 56,176 BP sites.

### Intron sequence-derived features

First of all, we define the regions between 55 nt upstream and 3SS of introns as “information region”, and define the regions between 50 and 11 nt upstream of 3SS as “target region”. Clearly, the information region of an intron includes the target region (50 nt~11 nt upstream) and flanking nucleotides. BPs in an intron sequence are characterized by the information region and target region. We extract several features from the information regions of introns, and attempt to predict BPs by using these features. Therefore, we introduce following features.

It was observed [15] that BPs have preference for “A” nucleotides. Since nucleotide types provide great signals for recognizing BPs, we adopt the sparse profile to represent the nucleotide preference. Four types of nucleotides (A, C, G and T) can be respectively represented by 4-bit vectors 1000, 0100 0010 and 0001. We replace nucleotides in the sequence with 4-bit vectors, and then represent the information region of an intron as a 220-dimensional (55 × 4) feature vector.

The dinucleotide “AG” is usually depleted between a BP and 3SS of an intron [15], and thus dinucleotide types can bring information for the BP identification. Four nucleotide types can form 16 dinucleotide types, and 16 dinucleotide types can be encoded as 4-bit vectors (AA:0000, AC:0001, CA:0010, AG:0011, GA:0100, AT:0101, TA:0110, CC:0111, CG:1000, GC:1001, CT:1010, TC:1011, GG:1100, GT:1101, TG:1110, TT:1111) respectively. By replacing continuous dinucleotides in a sequence with the corresponding bit vectors, we can represent the information region of an intron as a 216-dimensional (54 × 4) binary vector named dinucleotide profile.

The motifs are discovered to be useful for the human branchpoint recognition [8, 15]. A position weight matrix (PWM), also known as a position-specific weight matrix (PSWM) or position-specific scoring matrix (PSSM), is a commonly used to represent the motifs of biological sequence. Since motifs are very useful for the biological element identification, we consider to use motif information represented by PWM. First, information regions of training introns are scanned to generate nonamers which have BPs at 6th position, and we calculate a 20 × 9 PWM matrix based on these nonamers. Then, we scan each nucleotide (excluding the first five and last five) along the information region of an intron, we score the corresponding 9mer which has the nucleotide at 6th position by using PWM, and we finally obtain a 45-dimensional real-value vector named PSSM profile.

The Markov motif provides motif information in a different way [10, 15]. PWM takes nucleotides independently, while the Markov model can consider the dependency between nucleotides by using the Markov model. We calculate the Markov motif in several steps. First, we scan nonamers in information regions of training introns, and nonamers are categorized as positive nonamers (branchpoint at 6th position) or negative nonamers (non-branchpoint at 6th position). We calculate probabilities ${\left\{{P}_i\left({s}_i\right)\right\}}_{i=1}^9$, ${\left\{{P}_i\left({s}_i|{s}_{i-1}\right)\right\}}_{i=2}^9$ s_*i*_ = {*A*, *C*, *G*, *T*} based on positive nonamers. For an intron, each nucleotide (exclude the first five and last five, 45 in total) in the information region are scored, by calculating the positive score *P*_*positive*_(*s*) with $P\left(\mathrm{s}\right)={P}_1\left({s}_1\right)\prod_{i\in \left\{2,3,\cdots, 9\right\}}{P}_i\left({s}_i|{s}_{i-1}\right)$. Similarly, we compute probabilities based on negative nonamers, and then calculate the negative score *P*_*negative*_(s) for each nucleotide. The Markov motif score of a nucleotide is log(*P*_*postive*_/*P*_*negative*_). Finally, we can obtain a 45-dimensional Markov profile for an intron.

The polypyrimidine tract profile (PPT) contains three scores. The first one is the pyrimidine content between putative BP and the 3SS. The second is the distance to the closest downstream polypyrimidine tract. The third is the score of the closest polypyrimidine tract. For an intron, we calculate three scores for each nucleotide ranging from 55 to 10 nt upstream, and thus we can obtain a 135-dimensional PPT profile. Polypyrimidine tract profile is detailedly described in [10, 17].

In total, we have five intron sequence-derived features. Therefore, we discuss how to build prediction models by using these features.

### Multi-label learning methods

We describe the characteristics of introns by using feature vectors. Here, we have to consider how to describe the locations of BPs in intron sequences. Specifically, we represent BP sites in the target regions of the introns by *k*-dimensional binary target vectors, in which the value of a dimension is 1 if the corresponding site is a BP and otherwise the value is 0.

**Fig. 2:**
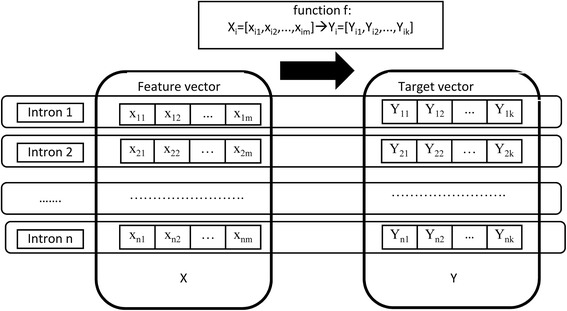
Multi-label learning for the branchpoint prediction

Given *n* introns, their feature vectors and target vectors are reformulated as the input matrix *X*_*n* × *m*_ and output matrix *Y*_*n* × *k*_, respectively. We aim to predict the locations of BPs for inputs introns, and predictions are multiple labels for 50 nt~11 nt upstream of 3SS, and the work is naturally taken as a multi-label learning task. The multi-label learning is different from the ordinary classification [18–20] which has one label, and it construct a model which simultaneously deals with multiple labels. For the BP prediction, the multi-label learning is to build the function *f* : *X*_*i*_ → *Y*_*i*_, where *X*_*i*_ = [*X*_*i*1_, *X*_*i*2_, ⋯, *X*_*im*_] and *Y*_*i*_ = [*Y*_*i*1_, *Y*_*i*2_, ⋯, *Y*_*ik*_] are the feature vector and the target vector of *i*th intron, *i* = 1, 2, ⋯, *n*. The flowchart of multi-label learning is demonstrated by Fig. 2. Considering the background, we have tens of thousands of instances (42,374 introns) and dozens of labels (40 labels) which represent 40 BP sites in the target regions of introns.

There are two types of multi-label learning algorithms [21–23]. One type is transformation methods, which transform the multi-label problem into a set of binary classification problems; the other is adaption methods which directly perform the multi-label classification. Transformation methods ignore correlation between labels, and adaption methods consider label correlation but need lots of times for training. An intron can have multiple BPs, and their locations may be correlated, and thus adaption methods are more suitable for our task. However, our problem has 40 labels, and most adaption methods can’t deal with so many labels because of the highly computational complexity. For the efficiency and effectiveness, we consider three matrix-based methods: partial least squares regression (PLS), canonical correlation analysis (CCA) and regularized canonical correlation analysis (LS-CCA) as the multi-label learning engines to handle the task, for these methods can deal with large-scale data in the reasonable time. We briefly introduce three methods in the following sections.

### Partial least squares regression

Partial least squares regression (PLS) is used to find the relations between two matrices [11]. Input matrix *X*_*n* × *m*_ and output matrix *Y*_*n* × *k*_ are respectively projected to *u*_*n* × 1_ and *v*_*n* × 1_ by *p*_*m* × 1_ and *q*_*k* × 1_. *u* = *Xp* and *v* = *Yq*, and the optimization objective is given by,

$$ {\displaystyle \begin{array}{c}\hfill \max \left\{{u}^Tv\right\}\hfill \\ {}\hfill \mathrm{Subject}\  \mathrm{to}:{\left\Vert p\right\Vert}_2=1,{\left\Vert q\right\Vert}_2=1\hfill \end{array}} $$

By using the Lagrange multiplier, we can solve the optimization problem, and know that *p* and *q* are respectively the eigenvector of largest eigenvalues of X^*T*^ *YY* ^*T*^ *X* and Y^*T*^ *XX* ^*T*^ *Y*, and then calculate *u* and *v*.

*X* and *Y* are reconstructed from *u* and *v* by *X* = *uc* ^*T*^ + *E* and *Y* = *vt* ^*T*^ + *F*; *Y* is reconstructed from *u* by *Y* = *ur* ^*T*^ + *G*. By using the least squared technique, we can obtain $c=\frac{X^Tu}{{\left\Vert u\right\Vert}_2^2}$, $t=\frac{Y^Tv}{{\left\Vert v\right\Vert}_2^2}$ and $r=\frac{Y^Tu}{{\left\Vert u\right\Vert}_2^2}$.

The residue *E* and *F* can be used as the new *X* and *Y*. We repeat *τ* times of above procedures to produce ${\left\{{p}_i\right\}}_{i=1}^{\tau }$, ${\left\{{q}_i\right\}}_{i=1}^{\tau }$, ${\left\{{u}_i\right\}}_{i=1}^{\tau }$, ${\left\{{v}_i\right\}}_{i=1}^{\tau }$, ${\left\{{c}_i\right\}}_{i=1}^{\tau }$, ${\left\{{t}_i\right\}}_{i=1}^{\tau }$ and ${\left\{{r}_i\right\}}_{i=1}^{\tau }$. $Y={u}_1{r}_1^T+{u}_2{r}_2^T+\cdots +{u}_{\tau }{r}_{\tau}^T+G$.

Let *U* = [*u*_1_, *u*_2_, ⋯*u*_*τ*_], *P* = [*p*_1_, *p*_2_, ⋯*p*_*τ*_], *R* = [*r*_1_, *r*_2_, ⋯*r*_*τ*_]. The prediction model is *Y* = *UR* ^*T*^ = *XPR* ^*T*^. For the new input *X*_*new*_, the output *Y*_*predict*_ = *X*_*new*_ *PR* ^*T*^.

### Canonical correlation analysis

Canonical correlation analysis (CCA) is to compute the linear relationship between two multi-dimensional variables [12]. Input matrix *X*_*n* × *m*_ and output matrix *Y*_*n* × *k*_ are respectively projected to *u*_*n* × 1_ and *v*_*n* × 1_ by *u* = *XP* and *v* = *Yq*, and the objective function is written as,

$$ {\displaystyle \begin{array}{c}\hfill \max \left\{{u}^Tv\right\}\hfill \\ {}\hfill \mathrm{Subject}\  \mathrm{to}:{\left\Vert u\right\Vert}_2=1\;\mathrm{and}\;{\left\Vert v\right\Vert}_2=1\hfill \end{array}} $$

By using the Lagrange multiplier, we can know that *p* and *q* are respectively the eigenvectors of (*X* ^*T*^ *X*)^−1^ *X* ^*T*^ *Y*(*Y* ^*T*^ *Y*)^−1^ *Y* ^*T*^ *X* and (*Y* ^*T*^ *Y*)^−1^ *Y* ^*T*^ *X*(*X* ^*T*^ *X*)^−1^ *X* ^*T*^ *Y*. *p*_1_ and *q*_1_ are eigenvalues of largest eigenvalues, u_1_ = *Xp*_1_ and *v*_1_ = *Yq*_1_ are first pair of canonical variables. Considering eigenvalues in descending order, we can obtain canonical variable pairs ${\left\{{p}_i,{q}_i\right\}}_{i=1}^{\tau }$, *τ* = min {*m*, *k*}.

Let *P* = [*p*_1_, *p*_2_, ⋯, *p*_*τ*_], *Q* = [*q*_1_, *q*_2_, ⋯, *q*_*τ*_].The prediction model is *Y* = *XPQ* ^−1^. For the new input *X*_*new*_, the prediction *Y*_*predict*_ = *X*_*new*_ *PQ* ^−1^.

### Regularized canonical correlation analysis

The canonical correlation analysis can be extended by introducing the regularization term, which control the complexity of the model. Therefore, Sun [13] proposed the regularized canonical correlation analysis (LS-CCA), and the optimization objective is,

$$ L\left(w,\lambda \right)=\sum_{j=1}^k\left(\sum_{i=1}^n{\left({X}_i{\left({W}_j\right)}^T-{Y}_{ij}\right)}^2+\lambda {\left\Vert {W}_j\right\Vert}_2^2\right) $$

Where *X*_*i*_ is the *i*th row of input matrix *X*, and *λ* > 0 is the parameter. The optimization problem can be rewritten as sub-problems,

$$ \mathit{\arg}\underset{W_j}{\mathit{\min}}\sum_{i=1}^n{\left({X}_i{W}_j-{Y}_{ij}\right)}^2+\lambda {\left\Vert {W}_j\right\Vert}_2^2 $$

For every *W*_*j*_, 1 ≤ *j* ≤ *k*, we can readily solve the problem by using the least angle regression algorithm. Let *W* = [*W*_1_, *W*_2_, ⋯, *W*_*k*_]^*T*^. The prediction model is *Y* = *XW*. For the new input *X*_*new*_, the prediction *Y*_*predict*_ = *X*_*new*_ *W*.

### Ensemble learning schemes for the branchpoint prediction

In machine learning, the primary goal of designing a prediction system is to achieve the high-accuracy performances. For a real problem, the instances are represented as features vectors, and then we construct prediction models based on feature vectors by using machine learning techniques. Several questions arise in the process of modeling. First, there are various features that describe characteristics of the instances, and how to make use of useful features is critical. The usual way of combining various features in bioinformatics is to concatenate or merge different feature vectors together, and we name the technique “direct feature combination”. Second, when you have several options for machine learning methods (classifiers), how to choose suitable methods is challenging. Researchers usually evaluate and compare classifiers to choose a suitable one, and then construct prediction models.

In recent years, the ensemble learning attracts great interests from bioinformatics community [24–31]. In this paper, we design the ensemble learning methods to combine various intron sequence-derived features and classifiers so as to build high-accuracy models for the BP prediction. Ensemble learning systems have two critical components, including base predictors and combination rules.

**Fig. 3:**
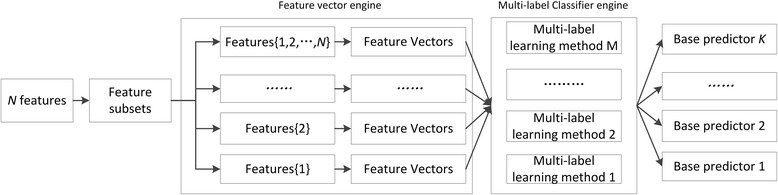
Constructing base predictors by combining feature subsets and multi-label learning methods

Base predictors are the primary component for the ensemble systems. Different base predictors can bring different information, and the diversity is of the most importance. To guarantee the diversity of base predictors, we make effects to make use of various features and various classifiers. Given *N* features, we have 2^*N*^ − 1 feature subsets, and merge the corresponding feature vectors to generate 2^*N*^ − 1 different kinds of features vectors. We combine these features vectors and *M* multi-label learning classifiers, and build *K* base predictors, where *K* = *M* × (2^*N*^ − 1). The construction of base predictor is illustrated by Fig. 3. In the branchpoint prediction, we have four sequence-derived features and three multi-label learning classifiers. Therefore, we can build a total of 45 base predictors.

Ensemble rules are the other component for the ensemble systems, which combine the outputs of base predictors. Designing an effective combination rule is very important for the ensemble learning system. The ensemble rule could be roughly divided into two types: trainable and non-trainable strategies. The trainable strategy integrates the outputs of base predictors, by building the relationship between the outputs of base predictors and real labels; the non-trainable strategy combines the scores of base classifiers as the final prediction, and the average scores are usually adopted. Given *K* base predictors: *P*_1_, *P*_2_, . . , *P*_*K*_, their prediction scores for a new input are *S*_1_, *S*_2_, ⋯, *S*_*K*_. Here, we respectively design the ensemble rules from the angles of the linear ensemble and non-linear ensemble.

The linear ensemble rule combines the prediction scores *S*_1_, *S*_2_, ⋯, *S*_*K*_ from base predictors with weights *w*_1_, *w*_2_, …, *w*_*K*_. The prediction of the ensemble system is the weighted average of all prediction scores, given by $\frac{\sum_{k=1}^K{w}_i\times {S}_k}{K}$. In the ensemble rule, the weights are free parameters and should be optimized. Weights are real positive values, and their sum should be equal to 1. Since we have dozens of base predictors, optimizing dozens of real weights is really a tough work. Here, the optimal weights are determined by the genetic algorithm. The genetic algorithm (GA) is a search approach based on the idea of biological evolution. In our design for weight optimization, we encode the candidate weights as chromosomes, and utilize GA optimization to search for the chromosome that maximizes the AUPR score on the data. The search start with a randomly initialized population, and the population updates with three operators: selection, crossover and mutation, and AUPR scores are used as the fitness scores. Finally, the optimal weights can be obtained.

The non-linear ensemble rule builds the nonlinear function *f* : ( *S*_1_, *S*_1_, …, *S*_*K*_) → {0, 1, which describes the relationship between the outputs of base predictors *S*_1_, *S*_2_, ⋯, *S*_*K*_ and real labels. The prediction by the ensemble learning system is given by *f* : ( *S*_1_, *S*_1_, …, *S*_*K*_). We have different functions for the nonlinear rules. Here, we use the logistic regression function $f\left(\ {S}_1,{S}_2,\dots, {S}_K\right)=\frac{1}{1+{e}^{-Z}}$, where z = *θ*_1_ *S*_1_ + *θ*_2_ *S*_2_ + ⋯*θ*_*K*_ *S*_*K*_ + *θ*_0_. The gradient descent technique can be used to determine the parameters *θ*_0_, *θ*_1_, …, *θ*_*K*_.

By using two ensemble rules, we design two ensemble learning systems for the branchpoint prediction. The first one is the genetic algorithm-based weighted average ensemble method, named “GAEM”; the other is the logistic regression-based ensemble method, named “LREM”.

## Results and discussion

### Evaluation metrics

In this paper, we evaluate methods on the benchmark dataset, by using 5-fold cross-validation (5-CV). In the 5-fold cross-validation, all introns are randomly split into five equal-sized subsets. In each fold, four subsets are combined as the training set, and the remaining subset is used as the testing set. The prediction model is trained on the introns in the training set, and then is applied to introns in the testing set. The training procedure and testing procedure are repeated, until each subset has been used for testing.

To test the performances of prediction models, we adopt several evaluation metrics, i.e. F-measure (F), precision, recall, accuracy (ACC), the area under the precision-recall curve (AUPR) and area under ROC curve (AUC). These metrics are defined as follows.

$$ {\displaystyle \begin{array}{c}\hfill ACC=\frac{TP+ TN}{TP+ TN+ FP+ FN}\hfill \\ {}\hfill Recall=\frac{TP}{TP+ FN}\hfill \\ {}\hfill Precision=\frac{TP}{TP+ FP}\hfill \\ {}\hfill F=2\times \frac{Precision\times Recall}{Precision+ recall}\hfill \end{array}} $$

Where *TP*, *TN*, *FP* and *FN* are the number of true positives, the number of true negatives, the number of false positives and the number of false negatives. Since non-BP sites are much more than BP sites, we take AUPR which considers both recall and precision as the most important metric. The cutoff which leads to the best F-measure is used to calculate accuracy (ACC), precision, recall and F-measure (F).

### Evaluation of intron sequence-derived features and multi-label learning methods

In BP prediction, we consider five intron sequence-based features and three multi-label learning methods. Here, we evaluate the classification abilities or usefulness of various features and different methods. We respectively use different methods to build individual feature-based models, and performances of models are indicators for the usefulness of features and methods. We adopt the default parameters for PLS (τ = 40), CCA (τ = 40) and LS-CCA (λ = 0.01). The individual feature-based models are evaluated under same experimental conditions.

**Fig. 4:**
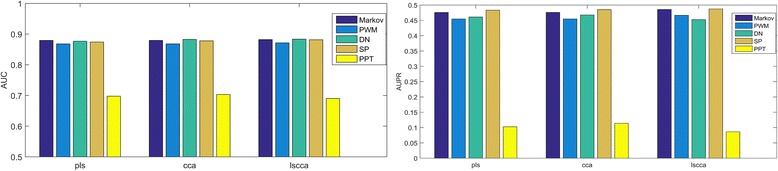
AUP scores and AUC scores of individual feature-based models. Markov: Markov motif profile, PWM: position weight matrix profile, DN: dinucleotide profile, SP: sparse profile, PPT: polypyrimidine tract, combination: combining all features

Figure 4 visualizes AUC scores and AUPR scores of different models, and thus we can compare different features and different methods. By using a same feature, different multi-label learning methods can produce similar performances; Markov motif profile, PWM and the dinucleotide profile have comparable performances when by using a same multi-label learning method, and the feature PPT produces the poorest performances.

The evaluation scores of prediction models are demonstrated in Table 1. The sparse profile produces the greatest AUPR score of 0.487. Markov motif profile, PWM and the dinucleotide profile yield the satisfying results, and PPT produces the poorest results in terms of all metrics. In general, LS-CCA leads to the better AURP score than PLS and CCA. Three methods produce similar results, but different methods may have advantages on different evaluation metrics.

**Table 1:** The performance of multi-label learning methods based on different features

Method	Feature	Recall	Precision	ACC	F	AUC	AUPR
PLS	Markov	0.508	0.478	0.961	0.473	0.879	0.476
	PWM	0.521	0.454	0.958	0.465	0.868	0.455
	DN	0.534	0.437	0.957	0.461	0.877	0.461
	SP	0.545	0.455	0.958	0.477	0.874	0.483
	PPT	0.574	0.098	0.787	0.170	0.698	0.103
CCA	Markov	0.529	0.461	0.959	0.472	0.880	0.476
	PWM	0.521	0.453	0.958	0.465	0.868	0.455
	DN	0.566	0.423	0.955	0.466	0.883	0.468
	SP	0.533	0.466	0.960	0.477	0.878	0.485
	PPT	0.488	0.118	0.844	0.182	0.703	0.114
LSCCA	Markov	0.502	0.501	0.963	0.482	0.882	0.486
	PWM	0.516	0.471	0.960	0.472	0.871	0.467
	DN	0.546	0.442	0.957	0.469	0.883	0.453
	SP	0.513	0.513	0.963	0.494	0.882	0.487
	PPT	0.472	0.085	0.790	0.129	0.690	0.086

Features describe different characteristics of branchpoints, and all features except PPT can lead to the high-accuracy prediction models. It is natural to combine these features to achieve better performances. However, different features share the redundant information, which may be the main concern in the feature combination. Here, we use a simple approach to test the negative impact of feature redundant information on the feature combinations. By using PLS as the baseline method, we combine features one by one according to the descending order of AUPR scores of individual feature-based models in Table 1. Based on different feature combinations, we merge corresponding feature vectors to build prediction models. As shown in Table 2, combining all features leads to the improved AUPR score of 0.494. For the feature combination models, we can also observe the improvements of the AUC scores and F-measure scores. In the combinations, SP can make the greatest contribution, and Markov can lead to the dramatic performance increase. But, the use of all features cannot necessarily lead to the best performances, and results show that the combination of SP, Markov, DN and PWM leads to best results.

**Table 2:** Performances of different feature combination models

Feature	Recall	Precision	ACC	F	AUC	AUPR
SP	0.545	0.455	0.958	0.477	0.874	0.483
SP + Markov	0.528	0.479	0.961	0.482	0.887	0.492
SP + Markov + DN	0.530	0.484	0.961	0.486	0.889	0.498
SP + Markov + DN + PWM	0.505	0.507	0.963	0.487	0.889	0.500
All	0.532	0.478	0.961	0.484	0.884	0.494

*Markov* Markov motif profile, *PWM* position weight matrix profile, *DN* dinucleotide profile, *SP* sparse profile, *PPT* polypyrimidine tract, *combination* combining all features

Moreover, we build binary classification models by using the same features (SP, Markov, DN and PWM), and compare binary classification models and multi-label classification models. We scan each nucleotide in the target region of an intron and obtain a nonamer which has the nucleotide at 6th position. We use the nonamer as the positive instance if the 6th nucleotide is a BP; otherwise, we use it as the negative instance. In this way, we have hundreds of thousands of binary instances for learning, and we adopt two popular and efficient binary classifiers: logistic regression and random forest to build prediction models. In the 5-fold cross validation, we make sure that the same training introns and testing introns are used for multi-label learning and binary classification learning in each split. The logistic regression model produces the AUC score of 0.878 and AUPR score of 0.324 when evaluated by 5-fold cross validation; the random forest model produces the AUC score of 0.842 and AUPR score of 0.329. The results show that the multi-label models can lead to better performances than the binary classification models, because the multi-label learning takes into account the correlation between putative BP sites.

Above studies demonstrate that features can provide useful information for the branchpoint prediction, but combining features effectively is difficult and need to be further studied. Therefore, four features and three algorithms are used to develop the final ensemble learning models for the branchpoint prediction.

### Performances of ensemble learning models

Given diverse intron sequence-derived features and several multi-label learning methods, we generate different feature subsets and merge corresponding feature vectors, and then adopt these methods to build base predictors. By using two ensemble rules to integrate outputs of base predictors, we develop two ensemble learning methods for the branchpoint prediction, namely the genetic algorithm-based weighted average ensemble method (“GAEM”) and the logistic regression-based ensemble method (“LREM”).

The genetic algorithm (GA) is critical for implementing GAEM. We set the initial population as 100 chromosomes. We implement GA optimization by using the Matlab genetic algorithm toolbox. The elitist strategy is used for the selection operator, and the default parameters are adopted for the mutation probability and crossover probability. GA terminates when the change on fitness scores is less than the default threshold or it meets the max generation number 100. We use the Matlab Statistics toolbox to implement the logistic regression, and then build the LREM models.

The results of GAEM and LREM on the benchmark dataset are given in Table 3. For comparison, performances of best individual feature-based models (built by LS-CCA) are also provided. GAEM and LREM produce the AUPR scores of 0.532 and 0.512 respectively. Clearly, ensemble learning models produce much better results than individual feature-based prediction models, indicating that both GAEM and LREM can effectively combine various features and different multi-label learning methods to enhance performances. In addition, LREM can produce better results than GAEM. The possible reason is that linear relationship in GAEM cannot deal with complicated data and nonlinear relationship in LREM is more suitable for our task.

**Table 3:** Performances of ensemble methods and best individual feature-based models

Feature	Recall	Precision	ACC	F	AUC	AUPR
Markov	0.502	0.501	0.963	0.482	0.882	0.486
PWM	0.516	0.471	0.960	0.472	0.871	0.467
DN	0.546	0.442	0.957	0.469	0.883	0.453
SP	0.513	0.513	0.963	0.494	0.882	0.487
LREM	0.529	0.537	0.965	0.515	0.904	0.532
GAEM	0.482	0.541	0.965	0.493	0.891	0.512

**Fig. 5:**
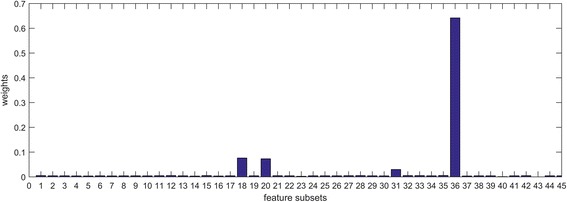
Weights in the GAEM model

In GAEM, the combination of feature subsets and multi-label learning methods are used to build base predictors, and the optimized weights are indicators for the importance of features and classification engines. There are 45 base predictors (15 feature subsets ×3 classifiers), and 45 weights are visualized in Fig. 5. We may draw several conclusions from the results. First, these optimal weights are different for base predictors, for they have different discriminative powers for the BP prediction. Second, the optimal feature subsets do not consist entirely of the highly ranked features. In Fig. 5, the 36th base predictors which are built based on Markov, PWM and SP by using LSCCA has the greatest weight.

**Fig. 6:**
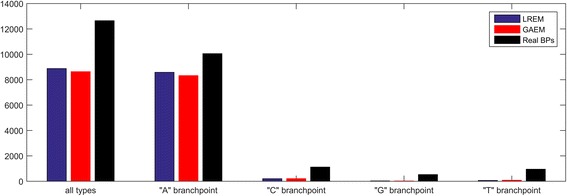
Correctly predicted branchpoints for all BPs and different types of BPs

Further, we design experiments to test the practical use of the genetic algorithm-based weighted average ensemble method (“GAEM”) and the logistic regression-based ensemble method (“LREM”). In the experiments, we randomly select 80% introns as the training set, and build the GAEM model and LREM model. Then, prediction models make predictions for the remaining 20% introns (8447). Prediction models predict the BP sites from 50 to 11 nt upstream of 3SS. Biologists give preference to most possible BP sites, and take wet experiments for verification. Therefore, we evaluate how many real BPs are identified. Here, we check top 3 predictions for each testing intron, and analyze the identified BPs. The statistics are shown in Fig. 6. LREM and GAEM can respectively identify 8878 BPs and 8635 BPs out of 12,650 real ones. The correctly identified BPs by two ensemble methods for different types of BPs are A: 8583, 8323/10054, C: 202, 208/1118, G: 28, 22/528, T: 65, 82/950. In general, LREM and GAEM can correctly find out 70.2% real BPs and 68.3% real BPs.

**Fig. 7:**
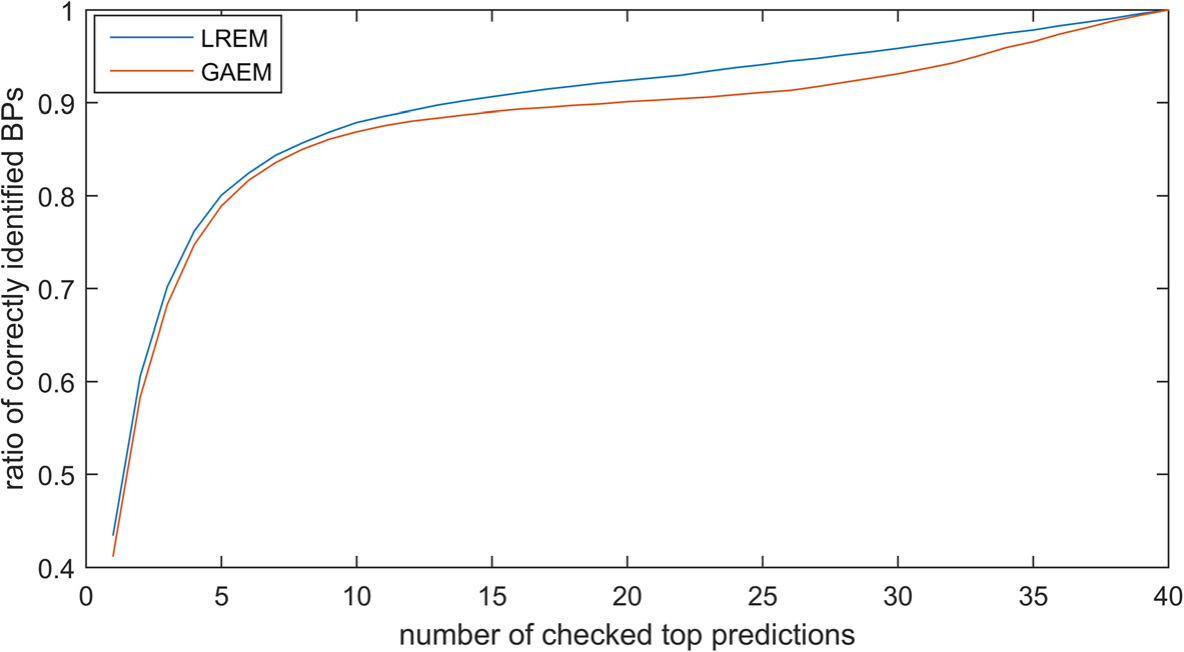
Ratio of correctly identified BPs versus number of checked top predictions

In addition, we evaluation the overall performances of LREM and GAEM in the independent experiments. For each intron, we check the top predictions, ranging from top 1 to top 40. We use the number of top predictions as X-axis and ratio of correctly identified BPs as Y-axis, and visualize the results in the Fig. 7. LREM and GAEM can identify more than 50% real BPs when only checking top 2 predictions for each intron, and they can find out most BPs from top 10 predictions. Thus, the proposed methods have the great recall scores in the independent experiments, and can effectively predict BP sites.

Therefore, the ensemble learning models GAEM and LREM can produce satisfying results for the branchpoint prediction.

### Comparison with other state-of-the-art methods

Although the BP prediction is an important work, only one machine learning-based method [10] named “SVMBPfinder” was proposed for the branchpoint prediction. First, SVMBPfinder defines a “TNA” pattern that has an “A” and a “T” two bases upstream. Then, SVMBPfinder scans 500 nt upstream to obtain all nonamers which have “TNA” in the central position, and takes conserved nonamers as the positive instances and others as negative instances. At last, SVMBPfinder uses Markov motif profile and PPT to encode nonamers, and then adopt SVM to build prediction models.

The source code of SVMBPfinder is publicly available. For fair comparison, we implement SVMBPfinder on our benchmark dataset, and make comparison under same conditions. SVMBPfinder only make predictions for the nonamers with TNA pattern and recognize the “A” BPs. However, according to our statistics on the benchmark dataset, BPs in TNA nonamers only take 53% of all BPs (34,120/63,371). We know that SVMBPfinder only identifies BPs from adenines, but ignores other BPs. In contrast, our methods can make predictions for all nucleotides located in 50 nt~11 nt upstream of introns. Here, we use two approaches to compare our methods and SVMBPfinder. One evaluation way (“local evaluation”) uses the predicted results and real labels for all TNA nonamers to calculate evaluation metric scores; in the other evaluation way (“global evaluation”), the smallest value of predicted scores for SVMBPfinder are assigned to non-TNA nonamers, and predicted scores and real labels for all nucleotides are adopted. Table 4 demonstrates that the ensemble methods LREM and GAEM can outperform SVMBPfinder in the global evaluation and local evaluation. More importantly, LREM and GAEM can predict TNA BPs as well as other types of BPs. Therefore, the proposed methods can produce high-accuracy performances, and has more practical use.

**Table 4:** Performances of our ensemble methods and the benchmark method

Evaluation	Local evaluation		Global evaluation	
Method	AUPR	AUC	AUPR	AUC
SVMBPfinder	0.502	0.576	0.362	0.729
LREM	0.524	0.906	0.532	0.904
GAEM	0.500	0.892	0.512	0.891

## Conclusion

Alternative splicing are biological processes that exert biological functions, and human splicing branchpoints help to understand the mechanism of alternative splicing. This paper is aimed to develop the computational method for the human splicing branchpoint prediction, by transforming the original work as a multi-label learning task. We investigate several intron sequence-derived features, and consider several multi-label learning methods (classifiers). Then, we propose two ensemble learning methods (LREM and GAEM) which integrate different features and different classifiers for the BP prediction. The experiments show two ensemble learning methods outperform benchmark methods, and produce high-accuracy results. The proposed methods are promising for the human branchpoint prediction.

## Abbreviations

5-CV: 5-fold cross validation
AUC: Area under ROC curve
AUPR: Area under precision-recall curve
BP: Branchpoint
GA: Genetic algorithm
